# Etymologia: Variola and Vaccination

**DOI:** 10.3201/eid1704.ET1704

**Published:** 2011-04

**Authors:** Nancy Männikkö

**Affiliations:** Author affiliation: Centers for Disease Control and Prevention, Atlanta, GA, USA

**Keywords:** Etymologia, variola, smallpox, pox, vaccine, vaccination

## *Variola *[və-ri′o-lə]

From the Latin for pustules or pox, possibly derived from varus, for pimple, or varius, for speckled. The earliest documented use of the word variola as a name for smallpox occurs in the 6th century, during the reign of the Byzantine emperor Justinian I. Referred to in the vernacular as simply “the pox” for many centuries, in the 16th century variola became known commonly as smallpox to distinguish the disease from syphilis, the great pox.

## Vaccination [vak′′sĭ-na′shən]

From the Latin vacca, for cow. English physician Edward Jenner coined the term vaccination in 1796 to describe inserting pus from cowpox lesions into open cuts on human patients to prevent smallpox. The term now refers to any immunizing procedure in which a vaccine is administered.

Sources: Hopkins DR. The greatest killer: smallpox in history. Chicago: The University of Chicago Press; 2002; Oldstone MB. Viruses, plagues, and history. New York: Oxford University Press; 1998; Tudor V, Strati I. Smallpox. Cholera. Tunbridge Wells (UK): Abacus Press; 1977.

